# Macro Photography with Lightsheet Illumination Enables Whole Expanded Brain Imaging with Single-cell Resolution

**DOI:** 10.15190/d.2021.12

**Published:** 2021-08-04

**Authors:** Chia-Ming Lee, Xuejiao Tian, Chieh Tsao, Peilin Chen, Tzyy-Nan Huang, Yi-Ping Hsueh, Bi-Chang Chen

**Affiliations:** ^1^Research Center for Applied Sciences, Academia Sinica, Taipei 11529, Taiwan; ^2^Brain Research Center, National Tsing Hua University, Hsinchu 30013, Taiwan; ^3^Institute of Molecular Biology, Academia Sinica, Taipei 11529, Taiwan; ^4^Institute of Cellular and Organismic Biology, Academia Sinica, Taipei 11529, Taiwan

**Keywords:** Macro photography, expansion microscopy, lightsheet microscopy, tissue clearing, whole-brain imaging.

## Abstract

Macro photography allows direct visualization of the enlarged whole mouse brain by a combination of lightsheet illumination and expansion microscopy with single-cell resolution.  Taking advantage of the long working distance of a camera lens, we imaged a 3.7 cm thick, transparent, fluorescently-labeled expanded brain. In order to improve 3D sectioning capability, we used lightsheet excitation confined as the depth of field of the camera lens. Using 4x sample expansion and 5x optical magnification, macro photography enables imaging of expanded whole mouse brain with an effective resolution of 300 nm, which provides the subcellular structural information at the organ level.

Volumetric imaging of whole organ structures at subcellular resolution helps scientists to understand the relationship between cell composition and organ function. Therefore, it is desirable to develop a microscopic tool to image large tissue samples with subcellular resolution. With recent advances in hydrogel-based tissue-clearing and tissue-expansion methods, it is now possible to obtain super-resolution images of tissue samples with sizes of hundreds of microns through conventional diffraction-limited microscopic techniques, such as lightsheet fluorescence microscope - a rapid volumetric technique with low photobleaching^1-5^, where microscopic objectives are used. In order to obtain brain-wide cellular distributions in an expanded brain^6^ with thickness of several centimeters, macro photography, a common close-up photography feature in the camera, is integrated with lightsheet illumination to image the whole brain with good optical sectioning capabilities. To visualize mouse brain structure, mice expressing nuclear tdTomato by crossing Ai75D mice and E2a-Cre mice (Jackson Laboratory, JAX stock #025106 and #003724, respectively) were used in this experiment. Mouse brain was fixed and collected at postnatal Day 14 (P14) (Figure 1A, inset).

**Figure 1:**
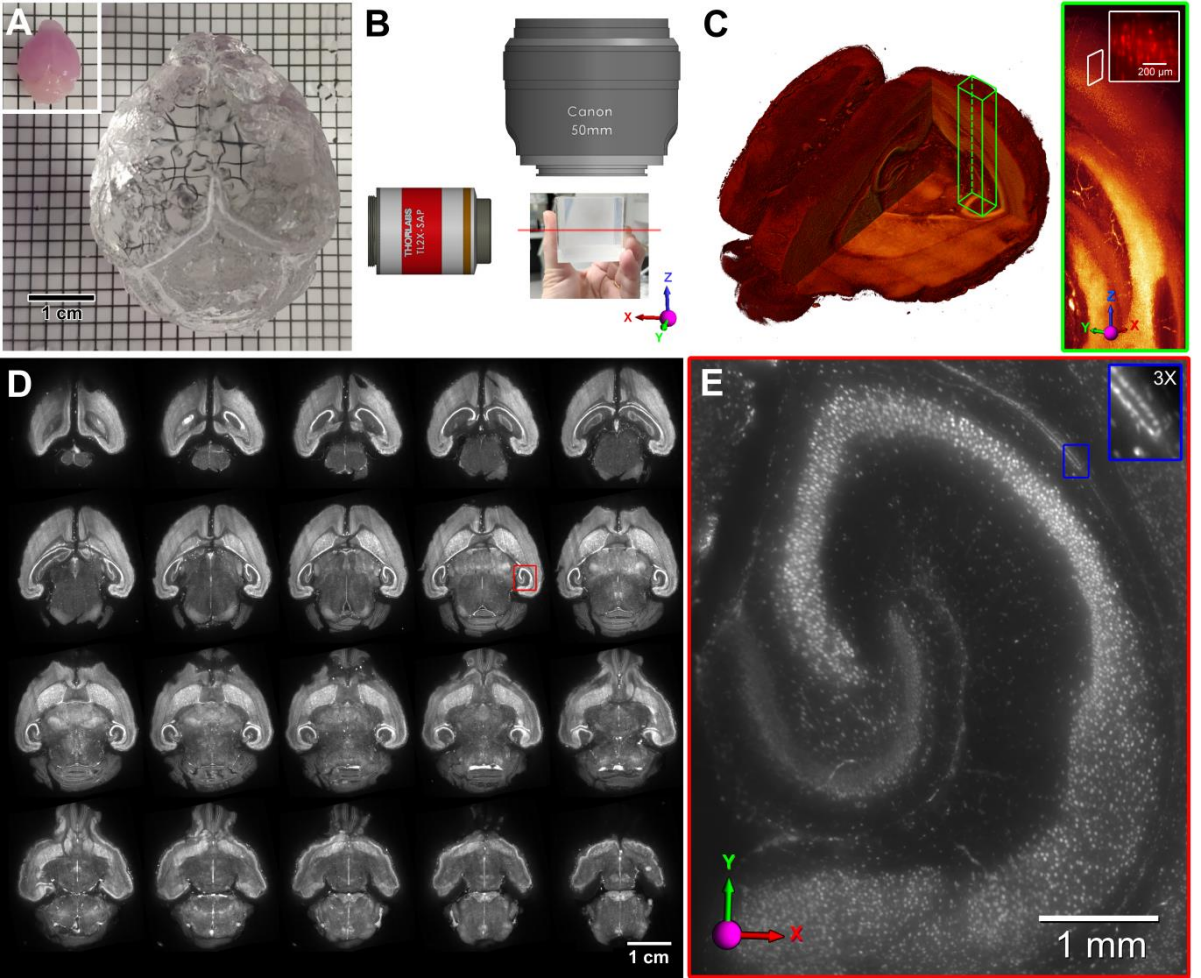
Macro lightsheet microscopy of an expanded whole mouse brain with single-cell resolution **A.** A whole mouse brain expressing nuclear tdTomato (upper left) was subjected to clearing and polymer hydrogel swelling protocols for expansion; **B.** Schematic layout of the major optical components for macro lightsheet microscopy, with the lightsheet formed by an excitation objective orthogonal to a Canon camera lens and the sample translated via a triple-axis (x,y,z) stage; **C.** Volume rendering of the expanded whole mouse brain. The green box represents a sub-volume of the brain image along the z-axis, with a zoomed-in image shown alongside in which the white box shows a xz view with single cells resolved in the z-direction; **D.** A series of expanded brain images captured at 700-µm intervals; **E.** Enlarged raw-resolution view of the area boxed in red in (D.), in which all nuclei of the hippocampus are labeled.

Through a combination of the passive CLARITY^7^ clearing method and a swellable hydrogel formulation^8^, we successfully expanded linearly the whole mouse brain by ~4.5-fold (∼100-fold volumetric expansion) (Figure 1A), where the sample volume was expanded from 1.25 x 1 x 0.6 cm^3^ to 5 x 3.75 x 2.4 cm^3^. Without mechanical sectioning, it is very challenging to image an expanded whole brain at single-cell resolution due to the limited working distance of the detection objective. To overcome this problem, we used a camera lens with sufficient working distance (~36 mm in the air) to allow optical sectioning via lightsheet illumination^9,10^ where a virtual thin lightsheet was generated by scanning a laser beam through a TL2X-SAP objective (Thorlabs, USA). The wide-field fluorescence images were captured through a reverse-mounted Single-lens Reflex Camera lens (SLR lens; EF 50 mm f/1.2L USM Canon Inc., Japan) with a 112 - 7 mm^2^ field-of-view at 1.25X to 5X magnification using a 4-megapixel sCMOS camera (ORCA-Flash4.0 V2, Hamamatsu, Japan) (Figure 1B). To image the whole intact brain, a glass chamber containing the expanded mouse brain with hydrogel was translated via a triple-axis stage. To facilitate high-magnification imaging, reverse-lens macro photography was used. With this setup, we were able to reconstruct the expanded whole brain in 3D in which all nuclei were labeled with red fluorescent tdTomato protein (Figure 1C), encompassing ~1 million 2Kx2K images at a voxel size of ~1.3 x 1.3 x 5 µm^3^. The effective resolution for an expanded mouse brain in our system is ~0.3 x 0.3 x 1 µm^3^ due to the ~4-fold expansion ratio. In Figure 1C, the green box represents a sub-volume along the axial direction of the camera lens, where individual cells can be resolved as shown in the white box in the inset. The selected optical slices of the expanded mouse brain are shown in Figure 1D, with an interval of 700 µm between contiguous sections. The zoomed-in image of the red box in Figure 1D of the hippocampus area is shown in Figure 1E, where a single-cell resolution was achieved laterally (blue box in Figure 1E) by macro photography with lightsheet illumination.

In summary, we have constructed a macro photographic system integrated with lightsheet illumination to reveal the three-dimensional cellular distribution in the whole brain with an effective resolution of 300 nm, where the samples were cleared and expanded.
